# The Nectarine Pit as a Cause for Small Bowel Obstruction and Perforation: A Case Report

**DOI:** 10.1155/2013/902943

**Published:** 2013-03-27

**Authors:** Mahmoud Al-Najjar, Thomas Arthur

**Affiliations:** ^1^Faculty of Health Sciences and Medicine, Bond University, University Drive, Robina, QLD 4226, Australia; ^2^Surgical registrar, Gold Coast Hospital, Southport, QLD 4215, Australia

## Abstract

Ingestion of a foreign body is a rare cause of small bowel obstruction. Ingested foreign bodies will usually pass without clinical sequelae, however on occasion can contribute to significant morbidity. Here we present an unusual case of small bowel obstruction and perforation as a result of accidental ingestion of a nectarine pit.

## 1. Introduction

Small bowel obstructions (SBOs) are a common presentation to hospital, accounting for around one in every seven surgical admissions [1]. It is estimated that 60% of these presentations will be due to adhesions [2]; however, it is important to be cognizance of the less common aetiologies when managing SBO. The ingestion of a foreign body is a rare cause of SBO. Here, we present an unusual case of SBO and perforation as a result of accidental ingestion of a nectarine pit.

## 2. Case Presentation

Mr. L. S. was a 67-year-old gent who presented to the emergency department of a large regional hospital with a one-day history of lower abdominal pain, nausea, and vomiting. He had been discharged two days previously after a three-day admission under a general medical team with vomiting and diarrhoea, presumed to be secondary to gastroenteritis.

The examination revealed a moderately obese gent with a distended abdomen and generalised abdominal tenderness. The patient was haemodynamically stable with no signs of peritonism. A small reducible umbilical hernia was present. On complete blood count, the patient was shown to have a leukocytosis of 20.1 × 10^9^/L, with a neutrophilia of 16.76 × 10^9^/L. His electrolytes were unremarkable; however, evidence of acute renal failure was apparent with a high creatinine of 236 umol/L and an eGFR of 24. Plain roentgenograms of the abdomen and chest showed multiple air fluid levels in the small bowel without dilatation, with no evidence of free air.

**Figure 1:**
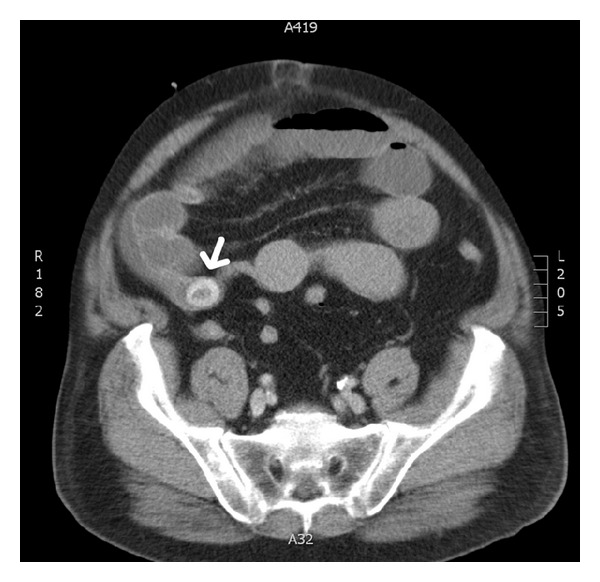
CT scan of the abdomen revealing 25 mm hyperdense lesion within the distal small bowel (white arrow).

Given the above findings, a CT scan was performed (Figure 1). On this scan, the distension of the jejunurm was evident, along with a hyperdense lesion in the distal small bowel around 25 mm in diameter. The large bowel was completely collapsed. The lesion was reported as a possible foreign body or gallstone ileus.

Mr. L. S. was taken to the operating theatre for a laparoscopy. This revealed a transition point in the distal ileum; however, the obstructing lesion was not identified. The operation was converted to an open laparotomy. The foreign body was discovered close to the ileocaecal junction and milked proximally. Enterotomy was performed, and a nectarine pit was retrieved.

**Figure 2:**
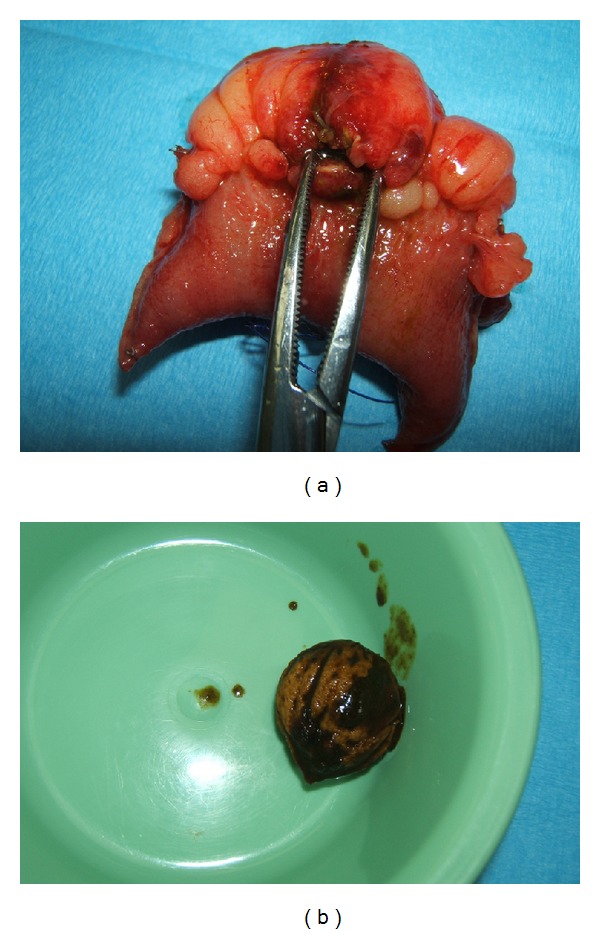
Small bowel perforation and offending pit.

The enterotomy was closed primarily. Further inspection of the small bowel revealed a perforation of the proximal ileum (Figures 2(a) and 2(b)). A short segment of ileum was resected, and a side-side anastomosis was performed with a linear stapler. After a 24-hour stay in ICU, the patient was discharged to the ward. The rest of his stay was unremarkable, and he was discharged on postoperative day 5. On later questioning, Mr. L. S. was unable to recall eating a nectarine in the days leading up to his admission; however, his wife stated that they had been eating nectarines over the last week.

## 3. Discussion

The ingestion of a foreign body is not an uncommon presentation to the emergency department. It is more often an affliction of the paediatric population; however, it does occur in the adult population and more commonly in edentulous adults [3]. Nonfood objects are more likely to be seen in adults with psychiatric disorders, developmental delay, and alcohol intoxication. Despite our patient having a previous cerebrovascular accident, there was no known swallowing or cognitive dysfunction as a result of this. Mr LS did have dentures, and this may have been contributory to his accidental ingestion.

The nectarine is a fruit of the tree *Prunus persica*, a native of China and southern Asia. Other fruits, such as apricots, persimmons, and plums, have previously been described in the literature as a cause of SBO, either through direct mechanical obstruction by the pit or through formation of a phytobezoar [4–6]. In this case, the nectarine pit appears to have caused a small bowel perforation in a position proximal to its final resting position near the ileocaecal valve. Physiological narrowings in the gastrointestinal system, such as the ileocaecal valve, are a common area for ingested foreign bodies and bezoars to become impacted. It is unusual that the pit caused a perforation proximal to its final resting place. It seems likely that this was a traumatic perforation from the sharp end of pit rather than an erosive process. 

Some larger series have estimated that the majority of foreign bodies, around 80–90%, will pass spontaneously; however, surgical intervention is required is some cases [7]. Mortality is rare, with a compilation of studies reporting no deaths amongst 852 cases in adults and 1 death in 2206 children [8]. Despite this, a significant morbidity can result, particularly, when there is a delay in the diagnosis, as there was in this case. Although SBO is a common presentation to hospital, it may not always have a common aetiology. It is important that clinicians maintain an open mind when managing SBO—knowledge of the patient's diet preceding admission may be essential in forming an accurate diagnosis.
